# Biomimetic properties and estimated in vivo distribution of chloroquine and hydroxy-chloroquine enantiomers

**DOI:** 10.5599/admet.929

**Published:** 2020-01-25

**Authors:** Klara L Valko, Tong Zhang

**Affiliations:** 1 Bio-Mimetic Chromatography Ltd, Business & Technology Centre, Bessemer Drive, Stevenage, Herts SG1 2DX UK; 2 Chiral Technologies Europe, Parc d’Innovation 160, Bd Gonthier d’Andernach CS 80140 67404 ILLKIRCH CEDEX France

**Keywords:** Protein binding, chiral separation, chloroquine and hydroxy-chloroquine, tissue binding, the volume of distribution

## Abstract

Chloroquine and hydroxy-chloroquine already established as anti-malarial and lupus drugs have recently gained renewed attention in the fight against the Covid-19 pandemic. Bio-mimetic HPLC methods have been used to measure the protein and phospholipid binding of the racemic mixtures of the drugs. The tissue binding and volume of distribution of the enantiomers have been estimated. The enantiomers can be separated using Chiralpak AGP HPLC columns. From the α-1-acid-glycoprotein (AGP) binding, the lung tissue binding can be estimated for the enantiomers. The drugs have a large volume of distribution, showed strong and stereoselective glycoprotein binding, medium-strong phospholipid-binding indicating only moderate phospholipidotic potential, hERG inhibition and promiscuous binding. The drug efficiency of the compounds was estimated to be greater than 2 % which indicates a high level of free biophase concentration relative to dose. The biomimetic properties of the compounds support the well-known tolerability of the drugs.

## Introduction

Chloroquine (CQ) and hydroxy-chloroquine (HCQ) are well established for the treatment of malaria, rheumatoid arthritis, and lupus. CQ was first synthesised in 1934 by Hans Andersag at Bayer in Elberfeld Germany, by modifying the chemical structure of quinine an anti-malarial natural alkaloid [1]. During the Second World War, when millions of soldiers were taking CQ for malaria prevention, its beneficial effects were discovered in the treatment of rheumatoid arthritis (RA) and systemic lupus erythematosus (SLE). When the resistance of CQ against malaria plasmids became apparent, the search for analogues resulted in the discovery of HCQ in 1945 [1]. The activity of CQ against viruses was first reported in 1972. by Shimizu *et al*. [2]. More than ten years ago, CQ was proposed as a broad-spectrum antimicrobial agent against bacteria, fungal and viral infections [3]. In December 2019, an outbreak of pneumonia of unknown aetiology was reported in Wuhan, China [4,5]. This disease problem was then declared as a pandemic by the World Health Organization (WHO) on the 11^th^ March 2020. The coronavirus was identified as the cause of this respiratory syndrome, and it was named SARS-CoV-2. The disease that it caused was named COVID-19. Since then, attention has been given to the use of CQ and HCQ as potential treatments for COVID-19.

The various mechanisms by which CQ and HCQ may be involved in their antiviral effects have been reviewed recently [6] as a result of increased interest due to the COVID-19 pandemic. Potential mechanisms include prevention of the virus entering the cells, altering virus replication, inhibiting autophagy and modulating the immune response. An interesting antiviral mechanism of CQ and HCQ is that they allow Zn ions to enter the cell and act as zinc ionophores [7]. Zinc ions exhibit anticancer and antiviral properties by altering the lysosome membrane permeability [8] and the viral RNA-dependent RNA polymerase enzyme and thus inhibit the virus replication process inside the cells.

**Figure 1.**
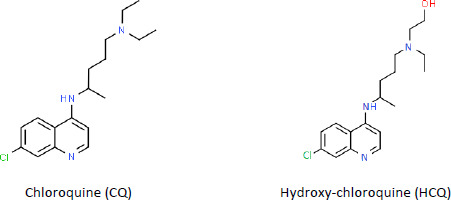
The chemical structure of chloroquine (CQ) and hydroxy-chloroquine (HCQ)

Figure 1 shows the chemical structures of CQ and HCQ. HCQ has an additional hydroxyl group that makes the molecule less lipophilic, reducing the severity of side effects and toxicity.

Both compounds are racemic mixtures, but it has been observed that the enantiomers behave differently *in vivo* regarding their pharmacokinetics [9] and metabolism [10]. It has been reported that the desethyl CQ excreted by men after treatment with racemic CQ is optically active [11] and that the renal clearance of the racemic CQ drug is a stereoselective process [9,12,13]. *In vitro* efficacy studies did not show any difference between the efficacy of the enantiomers [14], but *in vivo* (rat) studies have shown a greater efficacy for the (+)-(S)-CQ than the (-)-R-enantiomer [13,15,16]. More recent article [17] suggests chiral switches of enantiomers as more potent forms against Covid-19. It may be explained by the stereoselective disposition of CQ and HCQ *in vivo*. Several studies have already been published on the stereoselective blood and plasma protein binding of the two compounds using pure enantiomers [18-20] added to plasma and blood.

The application of chemically bonded chiral stationary phases (Chiralpak HSA and Chiralpak AGP) is a powerful tool for separating racemic mixtures [20]. It was found that the CQ enantiomers differ in their binding to plasma. The (+)-(S)-CQ has a higher binding to human serum albumin (HSA) than the (-)-(R)-enantiomer, whereas the latter bound more strongly to α-1-acid-glycoprotein (AGP) than the (+)-(S)-CQ. The *in vitro* human plasma protein binding of HCQ was also enantioselective: the binding of (+)-(S)-HCQ was almost 2-fold higher than (-)-(R)-HCQ (64 % against 37 %) [21]. Chromatographic separations of CQ and HCQ and their metabolites have been reported using Chiralpak AGP and Chiralpak AD HPLC columns [18,22], [23]. Other chromatographic methods have been reported for analytical and preparative separations of CQ and HCQ enantiomers [24,25].

Biomimetic HPLC properties can be used to estimate the *in vivo* distribution (volume of distribution, *V*_d_, the unbound volume of distribution, *V*_du_), drug efficiency and various tissue binding properties of compounds [26-28]. The HSA and AGP binding can be measured using Chiralpak HSA and Chiralpak AGP HPLC columns (Chiral Technologies Europe, France). In this way, a different estimation of the *in vivo* distribution and tissue binding for the different enantiomers can be obtained supposing they provided two requisite peaks from the chromatography, enabling property measurements.

The aim of this study was to measure the biomimetic properties of CQ and HCQ with special emphasis on the different properties of their enantiomers. The other aim was to rationalise the differences in the *in vivo* potency of the enantiomers that were not detected *in vitro*. A comparison can then be made of the estimated tissue binding and distribution data with the published *in vivo* distribution data. The significance of the differences in properties between the (R) and (S) enantiomers can then be evaluated.

## Experimental

Racemic CQ and HCQ of the highest purity were purchased from Merck (Sigma-Aldrich) and were dissolved in DMSO at a 1 mM concentration. The 1 mM solutions were diluted further to 0.1 mM concentration from which 10 μL was injected onto the different HPLC columns.

An Agilent 1100 series HPLC system equipped with an autosampler and diode array detector was used for the measurements.

### Measurements of lipophilicity at three pHs

Gemini NX C-18(2) 5 um columns with dimensions of 3 x 50 mm (Phenomenex Ltd. Macclesfield, UK) were used for the determination of the Chromatographic Hydrophobicity Index (CHI) and the CHIlogD values [26]. The flow rate was 1.0 mL/min, with starting mobile phases of 0.01 M formic acid (pH 2.6), 50 mM ammonium acetate adjusted to pH 7.4, and 50 mM ammonium acetate with pH adjusted to 10.5 to determine the lipophilicity of the compounds at acidic, neutral and alkaline pHs, respectively. An acetonitrile linear gradient was used from 0 to 100 %. The acetonitrile concentration reached 100 % in 3.5 min. The 100 % acetonitrile mobile phase was maintained for an additional 1 min before it was returned to 0 % at 4.7 min. The cycle time of the gradient run was 6 min, with an additional equilibration time of 1 min before the next injection. The error in the retention time measurements is ±0.005 min in general after repeated injections.

The set of calibration compounds gave an excellent straight line when their retention time data was plotted against the predefined CHI values listed in Table 1.

### Measurements of phospholipid-binding at pH 7.4 using an Immobilized Artificial Membrane (IAM) column

The phospholipid-binding was measured using an IAM PC.DD2 column with dimensions of 100 x 4.6 mm (Regis Technologies Inc., Morton Grove, IL, USA). The gradient retention times of the compounds were measured using a 50 mM ammonium acetate starting mobile phase with the pH adjusted to 7.4. The mobile phase flow rate was 1.5 mL/min. The acetonitrile gradient was applied from 0 min to 4.75 min to reach 90 %. The 90 % acetonitrile concentration was maintained for an additional 0.5 min (to 5.25 min), then returned to 0 % by 5.5 min. The cycle time was 6 min plus an additional 1 min equilibration time was applied while the injector prepared for the next injection. The gradient retention times were calibrated with the acetophenone homologues for which the CHI IAM values have been established using isocratic measurements [26]. The Chromatographic Hydrophobicity Index on the IAM column [CHI(IAM)] approximates to the acetonitrile concentration in the mobile phase when the compound elutes. CHI(IAM) values above 45 indicate strong phospholipid binding. Repeating the retention time measurements provided the error only in the third digit of the minute (±0.005).

### Measurements of plasma protein binding using Chiralpak HSA and AGP columns

**Figure 2.**
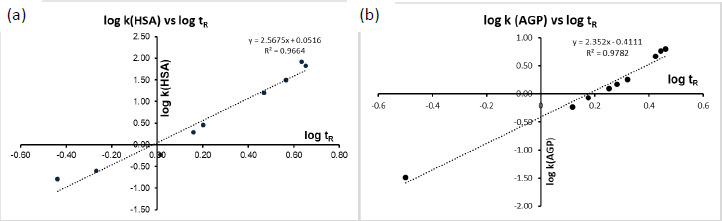
The calibration line obtained on the Chiralpak HSA (a) and Chiralpak AGP (b) columns by plotting the logarithmic value of the obtained retention times and the log *k* values derived from the literature % binding data [30,31]

The protein binding measurements were carried out on Chiralpak HSA and Chiralpak AGP columns with the dimensions of 3 x 50 mm with 5 um particle size stationary phase (Chiril Technologies Europe, France). The mobile phase was 50 mM ammonium acetate adjusted to pH 7.4 with a flow rate of 1.2 mL/min. The standard isopropanol (IPA) gradient up to 35 % was achieved in 3.5 min and then maintained for 1 min, before returning to 0 % at 4.7 min. The cycle time was 6 min with an additional 1 min re-equilibration time. The racemic warfarin showed separation of its enantiomers at retention times of 3.58 and 3.77 mins. The reproducibility of the retention time measurements was within ±0.01 min. The calibration set of compounds and their literature % binding data that were also converted to log *k* data are shown in Table 2. The racemic CQ and HCQ, however, did not show a separation of their enantiomers under such chromatographic conditions and therefore, other gradients and isocratic mobile phases were tested. Figures 2a and Figure 2b show the calibration lines obtained for the HSA and AGP binding, respectively.

### The 8 min, “slow gradient” method on the chiral HSA and AGP columns

To be able to separate the CQ and HCQ enantiomers, the IPA gradient was modified. From 0 to 4 min, the IPA concentration was raised from 0 to 15 %, and from 4 to 5 min the 15 % IPA was kept constant before it was reduced to 0 % at 5.2 min. The cycle time was 8 min plus an additional 1 min re-equilibration time. The same compounds and data that are listed in Table 2 were used to calibrate the retention times; however, warfarin, diclofenac and isopropanol could not be eluted under this condition.

### Isocratic measurements of CQ and HQ on Chiralpak HSA column

As the CQ and HCQ enantiomers were not separated using the longer IPA gradient, isocratic analyses were also carried out using a 1.2 mL/min flow rate with 5, 8 and 10 % IPA, respectively. Using 5 % IPA in the mobile phase, only paracetamol and propranolol eluted from the calibration set of compounds. When 10 % IPA was used warfarin, propranolol and nicardipine were eluted and their respective retention times were used to create a calibration curve for the calculation of the binding of CQ and HCQ from their obtained retention times using the same HPLC conditions.

### Measurements of human plasma protein binding by rapid equilibrium dialysis (RED)

The test compounds were prepared in 100 % pooled human plasma. The measurement was performed using equilibrium dialysis with the two compartments separated by a semi-permeable membrane. The plasma solution was added to one side of the membrane while buffer (pH 7.4) was added to the other side. The system was allowed to reach equilibrium at 37 °C. Compound concentration on both sides of the membrane was measured by LC-MS/MS, and the fraction of unbound compound calculated. Calibration standards were prepared in plasma and buffer. Test compound incubations were performed in triplicate. A control compound was included in each experiment. The solutions for each batch of compounds were combined into two groups (protein-free and protein-containing). Cassette analysed by LC-MS/MS using two sets of calibration standards for protein-free solutions (7 points) and protein-containing solutions (7 points). The experiments were carried out at Cyprotex Discovery Ltd (UK) and repeated at Sygnature Discovery Ltd (UK) laboratories using their generic LC/MS method. The unbound fraction (*f*_u_) is calculated from the concentration of the test compound in the protein-containing side *C*_p_ and the protein-free side *C*_f_ based on the formula:



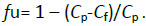



## Results and Discussion

The lipophilicity of compounds is an important physicochemical property that may explain the protein binding, the volume of distribution and other distribution properties of drug molecules. It is usually characterised by octanol/water partition (log *P*) and distribution (log *D*) coefficients. The log *P* refers to the neutral form of the molecule, while log *D* refers to the compound’s distribution between octanol and water at physiological pH (pH 7.4).

The calculated and measured physicochemical properties of CQ and HCQ are listed in Table 3.

It can be seen in Table 3 that the measured and calculated lipophilicity values vary a lot. In almost all cases, HCQ is less lipophilic due to the hydrophilic hydroxy group that also affects the basicity of this compound. Comparing the log *D* and log *P* values, it is apparent that a high percentage of both molecules are ionised (positively charged) at pH 7.4. The measured CHI log *P* may well be underestimating the lipophilicity as the p*K*a values are around 10. Therefore, the mobile phase applied pH 10.5 may not suppress the ionisation of both molecules completely, and the actual lipophilicity of the neutral form of these molecules can be much greater.

Table 4 shows the measured phospholipid and protein binding data of the two compounds obtained using the standard 6 min protocol [26].

It can be seen from Table 4 that HCQ has marginally weaker binding to phospholipids and plasma proteins. The enantiomers could not be separated on the chiral HSA and AGP stationary phases using the standard high-throughput protocol for biomimetic property measurements. The phospholipid-binding is in a medium-strong range. It has been reported that compounds that have CHI IAM values greater than 50 have the potential for promiscuous binding and causing phospholipidosis [34]. The hERG inhibition, which is an indicator for causing arrhythmia and heart condition, is also associated with lipophilic and positively charged compounds [35]. Positively charged compounds usually have stronger binding to IAM than isolipophilic compounds without the presence of positive charge [36]. However, the CHI IAM values of CQ and HCQ are below 50. The measured protein binding was greater than has been published [19], ranging from 46 to 74 % in patients with rheumatoid arthritis. Strong AGP binding was also observed, being approximately the same as the albumin binding.

However, the AGP concentration in the plasma ranges from 1 to 10 % while the albumin concentration in the plasma is around 60 %, being the most abundant protein. It is also well established that AGP prefers to bind to positively charged compounds since it has negatively charged sialic acids at its binding site [37]. The lysosomes inside the cell also contain glycoproteins that are negatively charged and attract positively charged compounds. The strong AGP binding of CQ and HCQ may explain the large variability of the plasma protein binding data as the AGP concentration depends on the disease state and it can increase significantly in inflammation, cancer and with age [38].

Table 5 shows the equations of the models that were used to make estimations of the *in vivo* properties of the drugs from the measured biomimetic HPLC data. Note, that the log *k* HSA and log *k* IAM data had to be transformed to log *K* HSA and log *K* IAM data as the measured log *k* data for acetophenone homologues up to octanophenone did not show linear correlation with their octanol water partition coefficients (log *P*) [39]. For the mechanistic model of the volume of distribution, we converted both the measured albumin and phospholipid binding data so that they showed linear correlation with the linear free-energy related log *P* values. Thus, the regression coefficients in linear regression analysis are meaningful and reflect to the physiological proportion of the albumin and phospholipids in the body. The estimated distribution and tissue binding data for CQ and HCQ were evaluated using the standard 6-min HPLC based measurements of biomimetic properties (CHIlog*D* at three pHs, log *k* HSA, log *k* AGP and log *k* IAM). They are based on the calibrated retention times obtained on C-18, IAM, Chiralpak HSA and Chiralpak AGP columns. The total plasma protein binding, lung tissue binding, drug efficiency, brain tissue binding and brain to blood ratio, cell partition coefficient and lung tissue binding have been calculated using the equations listed in Table 5 and are shown in Table 6.

It can be seen from Table 6 that both compounds have CAD-likeness values greater than 50, which indicates that both have phospholipidotic potential. It is known that chloroquine causes phospholipidosis both in animals and in human [40-42]. However, the phospholipidotic potential of HCQ is much less than CQ. Although the phospholipid-bindings (CHI IAM) were not very different, the greater difference in CAD-likeness values is due to the larger percentage of the positively charged form of CQ than in HCQ. It is obvious from the lower acid dissociation constant of HCQ and the smaller difference between the CHIlogP and CHIlogD values (see Table 3). It also implies that HCQ is less likely to cause hERG inhibition and promiscuous binding and in general, fewer side effects. Based on the measured data, the expected brain tissue binding did not show a significant difference between CQ and HCQ. On the contrary, the brain to blood ratio is smaller in HCQ than CQ due to the slightly stronger AGP binding of HCQ that keeps the compound in the plasma compartment.

Interestingly the expected volume of distribution based on the measured biomimetic properties is 6.9 and 5.8 L/kg for CQ and HCQ, respectively, which is not as high as over 100 L/kg as has been described in the literature [16,47-49]. The *V*_d_ of CQ was 116 to 285 L/kg in healthy volunteers while HCQ was reported to have a much higher volume of distribution at above 4000 L/kg [48]. Tett et al. [50], reported a range of steady-state *V*_d_ values for HCQ ranging from 2402 to 8346 L that are equivalent to 34.3 to119 L/kg. The volume of distribution was 5 to 7 times greater when the plasma concentration was considered instead of the blood concentration as the blood/plasma ratio was reported to be 5 to 7. The reported plasma protein binding value that was determined in 1986 was 66.6 % [20]; enantioselective plasma protein binding, albumin and AGP binding were also reported. The human plasma protein binding measurements were repeated using the rapid equilibrium dialysis method and the obtained average values were 52.6 % (±6.2 %) and 30.7 % (±11.3 %) for CQ and HCQ, respectively. The estimated plasma protein binding based on the biomimetic HPLC measurements was much stronger (95 % and 93 % for CQ and HCQ, respectively) than that published using *in vivo* data. At this stage, there is no explanation for the difference. This is the cause of the much lower estimated volume of distribution.

The AGP binding using equilibrium dialysis method was reported to be 48.4 % (59.9 and 34.9 % of the R and S enantiomers, respectively) for CQ and 34 % (29 and 41 % for the R and S enantiomer, respectively) for HCQ [51]. The albumin binding was only 38.6 and 40 % for CQ and HCQ, respectively, by the same author [51]. These values were quoted from earlier publications where the actual measurements were originally reported [52-54]. It was observed that under the standard IPA gradient conditions, both CQ and HCQ eluted between Carbamazepine and Nicardipine, being closer to Nicardipine, which binds to plasma proteins at 75 and 95 %, respectively. This explains why the HSA binding was calculated as being 87 and 83 % (see Table 3). The *DE*_max_ values reveal the free plasma concentration relative to the dose. Most marketed drug molecules have *DE*_max_ values of around 1 %. It can be seen that both CQ and HCQ have greater than 1 % drug efficiency, which indicates that a high free concentration can be achieved with a relatively low dose. The estimated lung tissue binding for both CQ and HCQ was weaker than their brain tissue binding. As mucus binding correlates very well with the AGP binding, strong mucus binding can be expected for both compounds. The cellular partition coefficients of both compounds are high (*K*_pcell_ above 7), which means a 7 to 8 times greater concentration than the intracellular concentration can be expected for both compounds.

**Figure 3.**
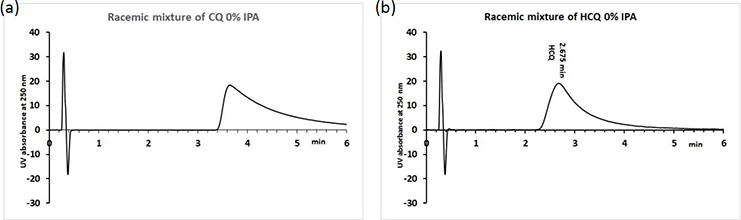
The chromatograms of CQ (a) and HCQ (b) on ChiralPak HSA column using 0 % IPA in the mobile phase in isocratic mode.

Unfortunately, our standard fast gradient protocols used for the HSA and AGP binding measurements did not allow the separation of the enantiomers of CQ or HCQ. This is also an indication that there are no significant differences in the biomimetic properties of each respective enantiomer. Therefore, new HPLC conditions were evaluated on the Chiralpak HSA and Chiralpak AGP column by increasing the gradient analysis time and reducing the maximum IPA concentration in the mobile phase. Slowing down the gradient to reach 10 and 20 % IPA did not result in the separation of the enantiomers on the HSA column, and both CQ and HCQ eluted after Carbamazepine (75 % plasma protein binding). The calculated HSA binding was 82.2 and 81.9 % for CQ and HCQ, respectively. Isocratic measurements using 5, 8 and 15 % IPA in the mobile phase resulted in 80 % binding when the relative retention times obtained using a partial set of calibration compounds were taken into consideration. No separation of the enantiomers was observed. Figure 3 shows the chromatograms of CQ and HCQ obtained on a ChiralPak HSA column using %IPA in isocratic mode. However, no separation of enantiomers was observed.

**Figure 4.**
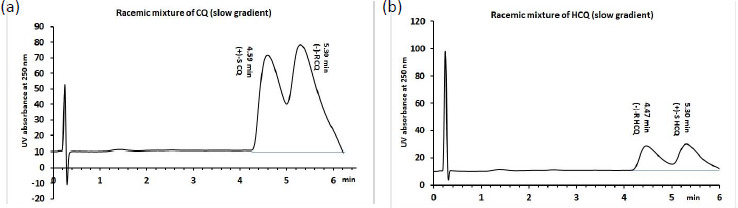
The chromatogram of racemic Chloroquine (CQ) (a) and hydroxychloroquine (HCQ) (b) obtained using the shallower gradient method (“slow gradient”) with 0 to 15 % IPA over 4.0 min and then maintained at 15 % for 1 min before returning to 0 % IPA on a Chiralpak AGP column (50 x 3 mm) with a 1.2 ml/min flow rate. The enantiomers are identified based on ref [51] that reported CQ (R) and HCQ (S) as the stronger binders to AGP.

When similar changes in the mobile phases were applied using the Chiralpak AGP column, excellent separations of both the CQ and HCQ enantiomers were observed albeit with a very short column size (L=50 mm) and fast flow rate (1.2 mL/min). It can be seen in Figure 4 that the separation of enantiomers occurred under the shallower gradient conditions. Still, no significant differences between the retention times were observed for CQ and HCQ confirming that the AGP binding of the two molecules is quite similar. The chiral separations were also obtained in the isocratic mode. However, the difference in binding between the enantiomers was only 3 to 4 %. Based on these measurements, the AGP binding is much stronger for both CQ and HCQ than previously reported. When the AGP binding is above 80 %, the compounds are expected to have variable efficacy in the clinic. It is because the AGP concentration changes in various disease states and it can alter the total plasma protein binding significantly, and that may cause a reduction of the free therapeutic concentration of the drugs.

Table 7 shows the % binding data obtained using a slow gradient (up to 15 % IPA). The protein binding measurements were repeated using isocratic conditions with 5, 8 and 15 % IPA in the mobile phase. Under such conditions, stronger binding compounds from the calibration mixture (warfarin, indomethacin and diclofenac) could not be eluted. The retention times of CQ and HCQ obtained when using 5, 8 and 15 % IPA on Chiralpak HSA and Chiralpak AGP columns are listed in Table 8.

It can be seen in Table 8 that the estimated % binding data using the retention time measured for the partial calibration set of compounds changes depending on the experimental conditions. The HSA binding ranged from 60 to 80 % but generally was much greater than the published albumin (HSA) and alpha-1-acid glycoprotein (AGP) binding data. This is because the retention times dependency on the IPA concentration was greater for the calibration set of compounds than for the CQ and HCQ. Table 9 shows the measured and estimated biomimetic properties of CQ and HCQ based on the data obtained when using the isocratic method on the Chiralpak HSA and Chiralpak AGP column (IPA concentration 15 %). Probably, CQ and HCQ do not bind to the hydrophobic site on the albumin but could bind to a specific area that is not affected strongly by the IPA concentration. The AGP binding was also much stronger using the biomimetic methodology that converts the % binding to the total plasma protein concentration where the AGP concentration is only 1 to 3 %. The difference can be explained by the different scaling used when expressing the % of the compound bound in plasma or to a single protein. However, when using the lowest IPA concentration in the mobile phase (5 %), the data obtained was between 77 and 87 % for the enantiomers of CQ and HCQ. The *in vivo* PK data also showed large individual variations in humans which suggests that these compounds may bind to a particular type of negatively charged proteins in plasma and in tissues that may have large concentration variations.

It can be seen that the estimated distribution data shown in Table 7 and Table 9 are very similar, and do not differ significantly despite the large differences in the % protein binding data. It is important to note that the % binding data are not linearly related to the protein binding partition coefficients. Still, they have a sigmoidal relationship with the log *k* and logarithmic retention time data. This means that small variations in the log *k* HSA and log *k* AGP values result in a large variation when expressed as percentage binding in the range of 30 to 80 %. In the stronger binding range, large variations in retention times will result in only a small per cent of change. The biomimetic protein binding data have been validated for differentiating between strong binders and are more accurate in that region [30].

## Conclusions

CQ and HCQ have gained renewed interest as potential cures for COVID-19 when used in combination with antibiotics and zinc. Biomimetic properties derived from biomimetic HPLC stationary phases have never been measured for these compounds. CQ and HCQ did not show extreme phospholipid-binding that would give the compounds a negative toxicity profile. CQ and HCQ bind to AGP very strongly and stereo-selectively, though there is no significant difference between CQ and HCQ in this regard. The stereoselective binding to AGP did not result in significant differences in the estimated total plasma protein binding, lung tissue binding and volume of distribution. The retention time measurements are very reproducible on the biomimetic columns, but the absolute values of the calculated binding data originate from the error of the calibration line fit. However, the order of the retention data is very reproducible. The *in vivo* distribution models include 0.5 to 1 log unit errors but again the rank order of the compounds is reproducible. The comparison between the estimated distribution properties and the properties published in the literature show great variations, however the biomimetic protein binding data were greater than the measured plasma protein binding data obtained by equilibrium dialysis. The aim of the study was to differentiate the binding of the various enantiomers and estimate the *in vivo* distribution of each enantiomer. It was found that the small differences in the protein binding data did not manifest itself in significant differences in the predicted *in vivo* distribution data, so this cannot explain the observed differences in the *in vivo* activity of the enantiomers. It was found that the drugs bind to glycoproteins that are present in humans in variable amounts from various disease states, which may explain the big variations observed in the *in vivo* distribution and protein binding data.

## Figures and Tables

**Table 1.** The CHI values of the calibration set of compounds at three pHs [29]. These values were obtained by fitting the isocratically determined CHI values and the gradient retention time values. The standard error ranged from 0.1 to 0.8 CHI values. The Chromatographic Hydrophobicity Index (CHI) approximates the acetonitrile concentration when the compound elutes. It can be converted to the octanol/water log *D* scale using CHIlogD = 0.0525*CHI -1.467.

Compound	CHI at pH 2.6	CHI at pH 7.4	CHI at pH 10.5
Theophylline	17.9	18.4	5.0
Phenyl tetrazole	42.2	23.6	16.0
Benzimidazole	6.3	34.3	30.6
Colchicine	43.9	45.0	43.9
Phenyl theophylline	51.7	51.2	51.3
Acetophenone	64.1	65.1	64.1
Indole	72.1	71.5	72.1
Propiophenone	77.4	77.4	77.4
Butyrophenone	87.3	87.5	87.3
Valerophenone	96.4	96.20	96.36

**Table 2.** The protein binding data of marketed drug molecules that were used to calibrate the retention times obtained on the chiral protein columns (Chiralpak HSA and Chiralpak AGP). The % binding data obtained by equilibrium dialysis were converted to log *k* data using log *k* = log (%binding/(101-%binding).

Compound name	% HSA	log *k* (HSA)	Retention times HSA (*t*_R_)	% AGP	log *k* (AGP)	Retention times AGP (*t*_R_)
Warfarin	97.9	1.5	3.6	83.2	0.7	2.6
Paracetamol	14.0	-0.8	0.4	3.2	-1.5	0.3
Nizatidine	20.4	-0.6	0.5	37.1	-0.2	1.3
Trimethoprim	37.6	-0.2	1.0	46.2	-0.1	1.5
Propranolol	66.6	0.3	1.4	86	0.8	2.8
Carbamazepine	75.0	0.5	1.6	65	0.3	2.1
Nicardipine	95.0	1.2	2.9	87	0.8	2.9
Indomethacin	99.5	1.8	4.5	56	0.1	1.8
Diclofenac	99.8	1.9	4.3	60	0.2	1.9

**Table 3.** Literature and calculated data for CQ and HCQ

Property	CQ	HCQ
ACD log *P* (calculated)	4.69	3.77
Chemaxon log *P* (calculated)	3.93	2.89
ACD log *D* (calculated)	1.74	1.96
Chemaxon log D (calculated)	0.88	0.33
Measured log *I* (ChemSpider [32, 33])	2.68-4.63	1.55 – 3.85
CHI log *D* (pH 7.4)	0.79	0.66
CHI log *P*	2.68	2.13
Chemaxon p*K*s (calculated)	10.32	9.76

**Table 4.** The measured phospholipid binding (CHI IAM), protein binding and estimated total plasma protein binding (PPB) based on the % HSA and % AGP binding and the lipophilicity of the compounds. The error of the values depends on the model error and the error obtained from fitting the calibration lines. The CHI IAM values are reproducible with ±3 CHI unit, the protein binding % error is larger at the middle range (± 10 %) and much smaller above 90 % binding (±0.1 %), the estimation of the total plasma protein binding includes the model error of ± 0.36 in log *k* PPB values.

Property	CQ	HCQ
CHI (IAM)	47	45.4
log *k* (IAM)	2.6	2.5
% HSA bound	87.6	84.9
log *k* HSA	0.8	0.7
% AGP bound	88.8	89.0
log *k* AGP	0.9	0.9
Estimated % plasma protein binding (PPB)	94.8	93.4
log *k* PPB	1.2	1.1

**Table 5.** The published and validated model equations that are used together with the measured properties to estimate the *in vivo* distribution behaviour of drugs. (abbreviations: IAM = Immobilized Artificial Membrane; HSA = Human Serum Albumin; *V*_d_ = volume of distribution; *V*_du_ = Unbound volume of distribution, *DE*_max_ = maximum drug efficiency; BTB = brain tissue binding; PPB = plasma protein binding, *f*_u_ = unbound fraction; *K*_bb_ = brain to blood partition coefficient; *K*_pcell_ = cell partition coefficient; LTB = lung tissue binding)

log *K* IAM [43]	= 0.29*e(0.026CHI(IAM)+0.42) +0.7
log *k* IAM [43]	= 0.046*CHI(IAM)+0.42
Phospholipidotic potential (CAD-likeness)	= CHI (IAM) + (CHI at pH 10.5 – CHI at pH 7.4)
log *K* HSA [43]	= elog *k*(HSA)
log *k* HSA [30]	= log (%HSAbound/(101- %HSA bound))
Estimated log *V*_d_ [43,44]	= 0.44*log *K* IAM -0.22*log *K* HSA – 0.62
Estimated log *V*_du_[27]	= 0.23*log *K* HSA +0.43*log *K* IAM -0.72
*DE*_max_ [45]	= 100/*V*_du_
log *k* BTB [31]	= 1.29*log *k* IAM+1.03*log *k* HSA-2.37
log *k* (PPB)[31]	= 0.98*log *k HSA*+0.19*log *k AGP*+0 .031*CHI log *D* 7.4-0.20
%BTB [31]	= 100*10log *k* BTB/(1+10log *k* BTB)
%PPB [31]	= 100*10log *k* PPB/(1+10log *k* PPB)
*f*_u_ BTB and PPB [31]	= (100-%BTB)/100 and (100 %-%PPB)/100
*K*_bb_ [31]	= *f*_u_PPB/*f*_u_BTB
log *K*_pcell_ [46]	= 1.1log *k* IAM-1.9
log *k* LTB [31]	= 0.49*log *k* PPB+0.34CHIlog *D*-0.069
% LTB [31]	= 100*10^log*k*LTB^/(1+10^log*k*LTB^)

**Table 6.** The estimated biomimetic properties of CQ and HCQ

Property	CQ	HCQ
Phospholipidotic potential (CAD-likeness)	82.8	73.6
%BTB	98.4	97.6
est log k BB	0.9	0.8
Brain to plasma	3.4	2.8
*V*_d_ (L/kg)	6.9	5.1
*V*_du_ (L/kg)	56.1	38.8
*DE*_max_%	1.8	2.6
% LTB	96.3	93.9
K_pcell_	8.7	7.3

**Table 7.** The measured and estimated biomimetic properties of CQ and HCQ based on the data obtained using the slower and shallower gradient (up to 15 % IPA).

Property	(+)-S CQ	(-)-R CQ	(+)-S HCQ	(-)-R HCQ
% bound HSA	82.2	82.2	81.9	81.9
% bound AGP	85.0	88.4	88.5	84.3
calc%PPB	90.6	91.1	91.2	91.7
CHI log *D*_2_	-1.2	-1.2	-1.3	-1.3
CHI log *D*_7.4_	0.8	0.8	0.7	0.7
CHI log *D*_10.5_	2.7	2.7	2.1	2.1
CHIlog *P*	2.7	2.7	2.1	2.1
CHI IAM	46.9	46.9	45.4	45.4
CAD-likeness	82.9	82.9	73.6	7367
%BTB	97.6	97.6	97.1	97.0
est log *k* BB	0.9	0.9	0.9	0.8
Brain to plasma	4.0	3.8	3.0	2.8
*V*_d_ L/kg	8.2	8.2	6.4	6.3
*V*_du_	45.7	45.8	35.3	35.1
*DE*_max_%	2.2	2.2	2.8	2.9
% lung tissue binding	95.5	95.6	93.1	93.3

**Table 8.** The retention times obtained for CQ and HCQ (enantiomers) using a 1.2 mL/min flow rate and 5, 8 and 15 % IPA on Chiralpak HSA and Chiralpak AGP columns. The calculated % binding data are also given in brackets. The retention times of Propranolol and Nicardipine, the two basic compounds from the calibration set, are also shown. The last two rows in the table are the data from reference [51] where the values were obtained using an equilibrium dialysis method (n/a means not applicable)

Compound/Cond.	CQ (R)	CQ (S)	HCQ (R)	HCQ (S)	propranolol (66 % PPB)	nicardipine (96 % PPB)
HSA 5 % IPA (min)	0.89 (60 %)	1.15 (68 %)	0.94 (61 %)	1.4 (73 %)	0.76 (54 %)	6 (96 %)
HSA 8 % IPA (min)	0.87 (64 %)	0.87 (64 %)	0.91 (66 %)	0.91 (66 %)	0.73 (57.6 %)	3.39 (95 %)
HSA 15 % IPA (min)	0.68 (80 %)	0.68 (80 %)	0.69 (80 %)	0.69 (80 %)	0.58 (66 %)	1.05 (95 %)
AGP 5 % IPA (min)	3.84 (86 %)	5.24 (90 %)	3.84 (86 %)	5.4 (90 %)	2.78 (85 %)	>8 min (>98 %)
AGP 8 % IPA (min)	2.53 (84 %)	2.55 (84 %)	2.95 (85 %)	3.85 (87 %)	1.73 (68 %)	5.63 (98 %)
AGP 15 % IPA (min)	2.20 (82 %)	2.00 (81 %)	1.98 (81 %)	2.23 (83 %)	1.58 (67 %)	5.0 (96 %)
% HSA bound [51]	30 %	54 %	50 %	29 %	n/a	n//a
% AGP bound [51]	59 %	35 %	29 %	34 %	n/a	n/a

**Table 9.** The estimated *in vivo* properties of CQ and HCQ enantiomers obtained from the data when the protein binding was obtained with the 15 % IPA isocratic conditions.

Property	(+)- S CQ	(-)- R CQ	(+)- S HCQ	(-)-R HCQ
% bound HSA	79.9	79.9	79.9	79.9
% bound AGP	82.0	81.0	85.1	87.3
calc% PPB	89.9	89.7	89.0	88.9
CHI logD_2_	-1.2	-1.2	-1.3	-1.3
CHI logD_7.4_	0.8	0.8	0.7	0.7
CHI logD_10.5_	2.7	2.7	2.1	2.1
CHIlogP	2.68	2.7	2.1	2.1
CHI IAM	46.92	46.9	45.4	45.4
CAD-likeness	82.89	82.9	73.6	73.6
%BTB	97.26	97.3	96.7	96.7
est log *k* BB	0.96	1.0	0.9	0.9
Brain to plasma	4.06	4.0	3.1	3.0
log *V*_d_	0.94	0.9	0.8	0.8
*V*_d_ L/kg	8.65	8.7	6.7	6.6
log *V*_du_	1.63	1.6	1.5	1.5
*V*_du_	43.01	43.0	33.5	33.3
*DE*_max_%	2.33	2.3	23.0	3.0
% lung tissue binding	96.05	95.1	92.6	92.7
